# Evaluation of a bespoke training to increase uptake by midwifery teams of NICE Guidance for membrane sweeping to reduce induction of labour: a stepped wedge cluster randomised design

**DOI:** 10.1186/s13063-017-2106-1

**Published:** 2017-07-27

**Authors:** Sara Kenyon, Sophie Dann, Lucy Hope, Paula Clarke, Amanda Hogan, David Jenkinson, Karla Hemming

**Affiliations:** 1 Institute of Applied Health Research, College of Medical and Dental Sciences, University of Birmingham, Edgbaston, Birmingham, B15 2TT UK; 2 Institute of Health & Society, University of Worcester, Henwick Grove, Worcester, WR2 6AJ UK; 3 Birmingham Women’s and Children’s NHS Foundation Trust, Mindelsohn Way, Birmingham, B15 2TG West Midlands UK; 4 Quality Assurance Team for Adult and Ante-natal/Newborn Screening Programmes, Midlands and East Region, 1st Floor, 5St Philip’s Place, Birmingham, B3 2PW UK; 5 Division of Health Sciences, Warwick Medical School, University of Warwick, Coventry, CV4 7AL UK

**Keywords:** Stepped wedge cluster randomised evaluation of training for community midwives

## Abstract

**Background:**

National guidance recommends pregnant women are offered membrane sweeping at term to reduce induction of labour. Local audit suggested this was not being undertaken routinely across two maternity units in the West Midlands, UK between March and November 2012.

**Methods:**

Bespoke training session for midwifery teams (nine community and one antenatal clinic) was developed to address identified barriers to encourage offer of membrane sweeping, together with an information leaflet for women and appointment of a champion within each team.

The timing of training session on membrane sweeping to ten midwifery teams was randomly allocated using a stepped wedge cluster randomised design. All women who gave birth in the Trusts after 39 + 3/40 weeks gestation within the study time period were eligible. Relevant anonymised data were extracted from maternity notes for three months before and after training. Data were analysed using a generalised linear mixed model, allowing for clustering and adjusting for temporal effects.

Primary outcomes were number of women offered and accepting membrane sweeping and average number of sweeps per woman. Sub-group comparisons were undertaken for adherence to Trust guidance and potential influence of pre-specified maternal characteristics. Data included whether sweeping was offered but declined and no record of membrane sweeping.

**Results:**

Training was given to all teams as planned. Analyses included data from 2787 of the 2864 (97%) eligible low-risk women over 39 + 4 weeks pregnant. Characteristics of the women were similar before and after training. No evidence of difference in proportion of women being offered and accepting membrane sweeping (44.4% before training versus 46.8% after training (adjusted relative risk [aRR] = 0.90, 95% confidence interval [CI] = 0.71–1.13), nor in average number of sweeps per woman (0.603 versus 0.627, aRR = 0.83, 95% CI = 0.67–1.01). No differences in any secondary outcomes nor influence of maternal characteristics were demonstrated. The midwives evaluated training positively.

**Conclusions:**

This stepped wedge cluster trial enabled randomised evaluation within a natural roll-out and demonstrates the importance of robust evaluation in circumstances in which it is rarely undertaken. While the midwives evaluated the training positively, it did not appear to change practice.

**Trials registration:**

ISRCTN14300475. Registered on 23 August 2016.

**Electronic supplementary material:**

The online version of this article (doi:10.1186/s13063-017-2106-1) contains supplementary material, which is available to authorized users.

## Background

In 2010–2011, approximately 21% of births in the UK were induced (NHS Maternity Statistics [1]). Induction is undertaken for a variety of indications, with post-term pregnancy being one of the most common. Induction of labour can have a negative impact on women’s birth experiences and is found by women to be more painful than spontaneous labour [2]. At the time the study was planned (2010–2011), women who laboured spontaneously had a Caesarean section (CS) rate of 11% and instrumental birth rate of 13%. In contrast, women who were induced had higher rates of CS (22%) and instrumental birth (17%). It is therefore important to do as much as possible to reduce the numbers of women requiring induction.

The National Institute for Health and Care Excellence (NICE) Guidance on Inducing Labour [3] reviewed the evidence on various relatively non-invasive methods of inducing labour, namely membrane sweeping, herbal supplements, acupuncture, homeopathy, castor oil, hot baths and enemas, sexual intercourse and breast stimulation. They concluded that there was insufficient evidence to recommend any of them other than membrane sweeping, which they recommend is undertaken to reduce induction of labour. See Table 1 for recommendations regarding membrane sweeping from NICE Inducing Labour Guideline. This Guideline was originally published in 2008 and regular Evidence Updates have not found need to update recommendations on the basis of published research, so the Guideline remains current and is due for review again in 2016 (http://www.nice.org.uk/guidance/cg70/documents/https://www.nice.org.uk/guidance/cg70). Membrane sweeping is also a NICE Antenatal Care Quality Standard (http://www.nice.org.uk/guidance/qs22/chapter/Quality-statement-12-Fetal-wellbeing-membrane-sweeping-for-prolonged-pregnancy).

**Table 1:** Recommendations regarding membrane sweeping from NICE Inducing Labour Guideline

Membrane sweeping involves the examining finger passing through the cervix to rotate against the wall of the uterus, to separate the chorionic membrane from the decidua. If the cervix will not admit a finger, massaging around the cervix in the vaginal fornices may achieve a similar effect. For the purpose of this guideline, membrane sweeping is regarded as an adjunct to induction of labour rather than an actual method of induction.
The Bishop score is a group of measurements made by doing a vaginal examination and is based on the station, dilation, effacement (or length), position and consistency of the cervix. A score of 8 or more generally indicates that the cervix is ripe, or ‘favourable’ – when there is a high chance of spontaneous labour, or response to interventions made to induce labour.
**1.3.1 Membrane sweeping** 1.3.1.1 Prior to formal induction of labour, women should be offered a vaginal examination for membrane sweeping. 1.3.1.2 At the 40- and 41-week antenatal visits, nulliparous women should be offered a vaginal examination for membrane sweeping. 1.3.1.3 At the 41-week antenatal visit, parous women should be offered a vaginal examination for membrane sweeping. 1.3.1.4 When a vaginal examination is carried out to assess the cervix, the opportunity should be taken to offer the woman a membrane sweep. 1.3.1.5 Additional membrane sweeping may be offered if labour does not start spontaneously.

### Assessment of current practice

An audit at Birmingham Women’s NHS Foundation Trust (BWNFT) had suggested that not all eligible women were offered membrane sweeping and maternity managers at Birmingham Heartlands Hospital (BHH) – part of Heart of England NHS Foundation Trust – also felt membrane sweeping was not done according to NICE Guidance in the maternity unit. Guidelines at BHH reflected those of NICE while BWNFT guidelines recommended sweeping of both nulliparous and multiparous women at 40 and 41 weeks. Collaboration between managers at the two Trusts and researchers at the University of Birmingham facilitated a robust evaluation of the effect of bespoke training for midwives to increase membrane sweeping to reduce induction of labour using a stepped wedge cluster randomised trial design.

## Methods

A stepped wedge cluster randomised trial was used as it was the intention of the healthcare providers that all midwifery teams (nine community and one antenatal clinic) receive the training. It was not possible to implement the training module over all teams concurrently and individual randomisation of midwives could not be used as the intervention was delivered to teams so contamination would be unavoidable. The stepped wedge design that was used is illustrated in Additional file 1: Figure S1. Data were collected for each team from 12 weeks prior to their team’s training being delivered and concluded at 12 weeks following the team’s training. The order in which each team received the training was randomised. In this pragmatic evaluation, we were limited by the number of midwifery teams in the area and so were not able to increase the number of clusters.

**Fig. 1:**
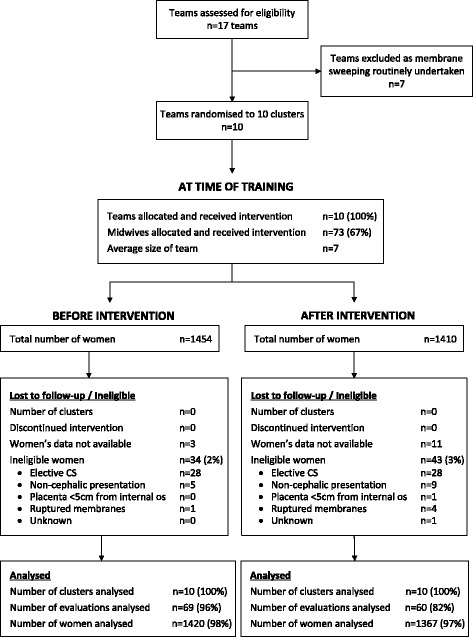
Consort flow diagram

Randomisation was performed in Stata, at a single point in time, by the study statistician (KH). Each of the ten midwifery teams were allocated a unique ID. These ten unique IDs were then randomly sorted to provide the order in which the teams would be trained. The teams were informed of their allocation date in sequential order once the previous team had set the date for training (a two-week period when training should be undertaken). Training took place between May and September 2012, and data were collected from 5 March 2012 to 26 November 2012, as shown in Fig. 1.

### Intervention

Midwives in the teams (nine community and one antenatal clinic) received the following: a generic interactive training package taking approximately 1 h containing evidence; practical tips to support practice; and a leaflet for women. The training was supported by written materials for the midwives.

A lead midwife (Champion) was identified in each team to be an expert for clinical queries and to train and remind staff.

A leaflet was developed for women entitled ‘Ways to reduce your need for induction of labour: membrane sweeping’, with input from the local Maternity Services Liaison Committee (service user group), with the intention that it would be given to women at 36/40 weeks gestation at their routine antenatal visit.

Training was led by the Practice Development Midwives/Consultant Midwives/Training Department in each Trust, supported by the NIHR Collaboration for Leadership and Applied Health Research and Care in Birmingham and the Black Country (CLAHRC BBC) researchers from the University of Birmingham.

As part of the development of the intervention one midwife from each team attended a group facilitated by CLAHRC researchers to discuss interventions they currently suggest to women or use to reduce induction of labour and to understand any barriers to the midwives sweeping women’s membranes. The concerns identified by the midwives included lack of time, unsuitable venue and the importance of maternal preparation. Some midwives also stated they were unsure of the technique and its effectiveness and expressed some reluctance to undertake membrane sweeping (painful for women and do not like to interfere with nature).

### Inclusion/exclusion criteria

All the midwifery teams were included in Trusts/Units where membrane sweeping was felt not to be done according to NICE and Trust Guidance. The number of clusters (i.e. teams) included was pre-determined based on teams to whom the Trusts had decided to roll out the training package.

Eligible women included all those who gave birth over 39 + 3 weeks at BWNFT or BHH within the study period (March to November 2012). Women were included who gave birth after 39 + 3 weeks (rather than over 40 weeks) as it is plausible that women were not swept on exactly the correct day (i.e. 40 or 41 weeks), so a sweep from 39 + 4 to 40 + 3 was considered a sweep at 40 weeks and 40 + 4 to 41 + 3 was considered a sweep at 41 weeks. Women were excluded if they were from outside the area or had an elective CS.

### Outcomes

The primary outcomes were the proportion of women offered and accepting a membrane sweep and the average number of sweeps per woman.

Secondary outcomes included onset of labour, mode of birth and adherence to Trust guidance. NICE guidance recommends that nulliparous women are offered membrane sweeping at 40 and 41 weeks and multiparous women at 41 weeks and BHH had adopted this. At BWNFT, Trust Guidance recommended all women were swept at 40 and 41 weeks regardless of parity. Information was also collected on sweeps offered but declined, where no record of membrane sweeping was found in the maternity notes, and the location of the sweep (community or hospital). If sweeping was abandoned, the reason was collected (e.g. cervical os closed, unable to reach, unable to sweep).

Planned subgroup analysis included whether the numbers of women having a membrane sweep was influenced by maternal age (<20 years and > 35 years), parity (nulliparous and multiparous), ethnicity (from antenatal notes), body mass index (BMI) (<18 kg/m^2^ and > 35 kg/m^2^) or Index of Multiple Deprivation (IMD) based on postcode.

Training was evaluated by the midwives using a questionnaire both immediately and six months afterwards.

### Data collection

Women were identified for inclusion in the analysis by the individual Trusts’ electronic systems and were included if they gave birth after 39 + 3 weeks gestation within the study period. It was planned that the data on sweeping status would form part of the mandatory electronic data collected within each Trust and that these data would be collected from the antenatal notes by the midwife entering birth outcome data onto the Trusts’ electronic systems. Information would be recorded as to whether the woman was offered sweeping, whether she accepted and at what gestation. However, this did not prove possible within the study time at BWNFT, so pseudo-anonymised data were extracted from the handheld antenatal records of 2864 women and entered onto a bespoke database. At BHH the mandatory electronic data collection began part way through the study (15 June 2012) and pseudo-anonymised data were transferred electronically to the University of Birmingham after that date. Prior to this, all data were extracted from the handheld maternity notes.

Planned data cross-checking with source notes of the electronically-transferred data on a random 20% of women found inaccuracies in 30% of data, so data on membrane sweeping, ethnicity, BMI, postcode and midwifery team were manually extracted from the handheld maternity notes for all women.

Systems were agreed with the Research and Development (R&D) Departments, which ensured only pseudo-anonymised data were transferred to and stored by the University of Birmingham. Data were extracted by members of the University team holding Research Passports and with permission to do so. All data were given a unique study specific number and only that required was extracted for the agreed analysis. Blinding to intervention period was not possible but personnel extracting data were not aware of individual team training dates.

### Sample size

From Hospital Episode Statistics, we estimated that there would be approximately 12 births after 40 weeks gestation per week in each team. Birth data were collected for each team from 12 weeks prior to training until 12 weeks following training. Given this fixed sample size, we determined what difference in the primary outcome (proportion of women swept) would be detectable with 80% power. We did not make allowances for the co-primary outcomes as these two outcomes are highly correlated.

The calculation depended upon both the current proportion of women being swept and the magnitude of intra-cluster correlation coefficient (ICC) between the proportions of women swept in each of the midwifery teams. Estimates of ICC would ideally come from other similar studies but, in the absence of such evidence, we were guided by a review of estimates of ICCs which found that their values are typically in the range of 0.02–0.1 [4]. A small audit suggested that of those eligible for sweeping, 32% of nulliparous women and 57% of multiparous women were currently being swept.

Methods described in Hussey and Hughes [5, 6] were followed to determine power and implemented using the Stata function [7]. It was estimated that at 5% significance (two-tailed) and 80% power, for ICCs in the range of 0.02–0.1 and for baseline event rates of 20–60%, the study would have power to detect around a 10% absolute increase in proportion of women being swept. This was an increase felt to be clinically worthwhile.

### Analysis

The participants’ characteristics were summarised using appropriate summary statistics, grouping them by whether they gave birth before or after the training session. These characteristics included the woman’s parity, ethnicity, BMI and IMD based on postcode as well as the Trust caring for her. Teams were classified as being exposed to the intervention the week after the team underwent the training and births during these transition weeks were not included. The trial was well balanced on all characteristics (Table 2) and so no adjustment was made for patient level characteristics in the outcome analysis.

**Table 2:** Participant baseline characteristics

Baseline characteristics	Before training (*n* = 1420)	After training (*n* = 1367)
Women’s age (years)		
Median (IQR)	29 (25–32)	29 (25–33)
< 20	78 (5.5%)	81 (5.9%)
20–35	1126 (79.3%)	1074 (78.6%)
> 35	216 (15.2%)	212 (15.5%)
Parity		
Nulliparous	850 (59.9%)	793 (58.0%)
Multiparous	569 (40.1%)	574 (42.0%)
Ethnicity		
Africa	89 (6%)	81 (6%)
Asia – South	442 (31%)	427 (31%)
Asia – Other	16 (1%)	19 (1%)
Caribbean	60 (4%)	57 (4%)
European – Britain	642 (45%)	619 (45%)
European – Other	65 (5%)	55 (4%)
Middle East	43 (3%)	47 (3%)
Other	60 (4%)	42 (3%)
Unknown	3 (0%)	20 (1%)
BMI (kg/m^2^)		
≤ 18	57 (4.0%)	54 (4.0%)
19–34	1254 (88.3%)	1199 (87.7%)
≥ 35	108 (7.6%)	112 (8.2%)
Index of multiple deprivation from postcode		
Quintile 1	911 (64.2%)	855 (62.5%)
Quintile 2	244 (17.2%)	253 (18.5%)
Quintile 3	191 (13.5%)	158 (11.6%)
Quintile 4	52 (3.7%)	67 (4.9%)
Quintile 5	20 (1.4%)	32 (2.3%)
Trust		
BWNFT	926 (65%)	871 (64%)
BHH	494 (35%)	496 (36%)

Note we exclude those ineligible for sweeping and those delivering within the training transition period. Percentages are of the total and include any women with missing data on that variable

*IQR* interquartile range

The primary aim of the study was to evaluate whether there was a difference in the proportion of women being swept in the 12-week period before and after the training session (intervention). To this end, we fitted a mixed effects Poisson regression model, using robust standard errors to account for the misspecification of the variances [8]. We included, as explanatory variables, the treatment exposure (before or after training, as a fixed effect), the midwifery team (as a random effect, accounting for the clustering) and calendar time (as a fixed effect). The treatment effect is reported as the adjusted relative risk (aRR) of being offered and accepting a sweep. The other primary outcome (number of sweeps) was also analysed by a mixed effects Poisson regression model, with the same explanatory variables. The treatment effect is the adjusted incidence rate ratio (aIRR) of having one extra sweep after the intervention compared to before it. The secondary outcomes were binary and were also analysed using Poisson models and, again, reporting RRs. For the analysis of subgroups, the same Poisson regression model was applied to a subset of the data containing the participants that belonged to that subgroup.

All analyses were carried out in duplicate, independently, to verify the results (KH and DJ). Results reported were carried out in R, although the independent verification was carried out in Stata 12. Comparisons will be considered significant at the 5% level and so 95% confidence intervals (CI) are reported throughout.

## Results

Training was given to all the midwifery teams as planned, with the majority of team members being present (73/108 [67%]). There were ten midwifery teams (nine community and one antenatal clinic) that included an average of ten midwives. The average size of team varied between the Trusts (BWNFT 14 and BHH 7).

Of the 2864 women identified by the Trusts as potentially eligible for inclusion, 2787 were included in the analysis (1420 women before training and 1367 after). Thirty-four women before and 43 women after the training were ineligible (Fig. 1) as seen in the CONSORT flow diagram. Data were not available for 14 women (three before the intervention and 11 after) and so they could not be included in the analysis. Membrane sweeping was offered and refused by 6% of women (Table 3). The characteristics of the women before and after training were similar (Table 2).

**Table 3:** Primary and secondary outcomes, sub-group by trust and process outcomes

	Before training (n =1417)	After training (n = 1356)	RR (95% CI)	*P* value
Primary outcomes				
Women offered and accepting membrane sweeping^a^	629 (44.4%)	634 (46.8%)	0.90 (0.71–1.13)	0.37
Mean average (SD) number of membrane sweeps per woman	0.603 (0.795)	0.627 (0.787)	Rate ratio: 0.83 (0.67–1.01)	0.068
Secondary outcomes				
Onset of labour^b^				
Induced	323 (22.8%)	328 (24.2%)	1.04 (0.80–1.34)	0.77
Mode of birth^c^				
Instrumental	235 (16.6%)	233 (17.2%)	1.06 (0.75–1.48)	0.75
Emergency CS	187 (13.2%)	177 (13.1%)	0.89 (0.63–1.26)	0.52
Sub-group by Trust				
BWNFT – Adherence to Trust guidance				
All women swept at 40 weeks (39 + 4 – 40 + 3)	245/921 (26.6%)	253/868 (29.1%)	0.91 (0.64–1.29)	0.596
All eligible women swept for a second time at 41 weeks^d^ (40 + 4 – 41 + 3)	78/504 (15.5%)	62/509 (12.2%)	0.75 (0.41–1.36)	0.339
BHH – Adherence to Trust (NICE) guidance				
Nulliparous women swept at 40 weeks (39 + 4 – 40 + 3)	47/174 (27.0%)	59/173 (34.1%)	1.81 (0.84–3.92)	0.131
All eligible nulliparous women swept for second time at 41 weeks^c^ (40 + 4 – 41 + 3)	10/80 (12.5%)	17/88 (19.3%)	2.28 (0.59–8.87)	0.232
Multiparous women swept at 41 weeks (40 + 4 – 41 + 3)	38/152 (25.0%)	46/160 (28.8%)	0.78 (0.32–1.88)	0.574
Process outcomes				
Sweeps offered but declined	80 (5.6%)	97 (7.2%)		
No record of sweeping	708 (50.0%)	625 (46.1%)		
Reason if abandoned				
Os closed	30 (4.8%)	28 (4.4%)		
Unable to reach	38 (6.0%)	20 (3.2%)		
Unable to sweep	50 (7.9%)	42 (6.6%)		
Other	13 (2.1%)	9 (1.4%)		
Location of sweep				
Community	400 (63.6%)	431 (68.0%)		
Hospital	227 (36.1%)	195 (30.8%)		

RRs are estimated using a generalised linear mixed model and are adjusted for clustering and underlying temporal trends

^a^The estimated ICC (95% CI) was 0.060 (0.000–0.118) estimated using a one-way analysis of variance on the proportions scale

^b^For onset of labour, the risk of being induced compared to spontaneous and not labouring combined was compared before and after training

^c^For mode of birth, the risk of instrumental birth compared to SVB and CS combined was compared before and after training. Separately, emergency CS was compared to SVB, instrumental and elective CS combined, before and after training

^d^Eligible women: pregnant at 41 + 3 weeks

There was no evidence of any differences in the primary outcome of numbers of women being offered and accepting membrane sweeping before and after training (44.4% versus 46.8%, aRR = 0.90, 95% CI = 0.71–1.13), nor in the average number of membrane sweeps being undertaken per woman (0.603 versus 0.627, aRR = 0.83, 95% CI = 0.67–1.01) (Table 3). RRs are adjusted for clustering and underlying temporal trends. There was no evidence of any differences in either secondary outcome, onset of labour or mode of birth (Table 3).

Trust-specific results for BWNFT found no evidence of differences in the primary outcome of numbers of women being offered and accepting membrane sweeping before and after training (47.4% versus 51.8%, aRR = 0.87, 95% CI = 0.67–1.14). However, the average number of membrane sweeps being undertaken per woman had significantly decreased (0.660 versus 0.701, aRR = 0.71, 95% CI = 0.55–0.90) (Table 3). Improvement in adherence to Trust Guidance was not seen between the two periods (Table 3).

Trust-specific results for BHH found no evidence of differences in the primary outcome of numbers of women being offered and accepting membrane sweeping before and after training (38.7% versus 37.7%, aRR = 1.17, 95% CI = 0.76–1.81) nor in the average number of membrane sweeps being undertaken per woman (0.497 versus 0.493, aRR = 1.32, 95% CI = 0.89–1.95) (Table 3). Improvement in adherence to Trust guidance was not seen between the two periods (Table 3).

No differences were seen in any other outcome for either Trust.

The comparison of the effect of selected characteristics on the intervention training demonstrated no individual effect of maternal age, parity, ethnicity, BMI or IMD from postcode (Additional file 2: Table S1).

Response rates to the training questionnaires were good: 69/73 (95%) immediately following training and 60/73 (82%) six months after training. Overall evaluation of training showed knowledge of the evidence and current NICE Guidance regarding membrane sweeping was high before training (average 4/5), improved (to 5/5) immediately after training and reduced slightly (to 4/5) at six months after training. Of the midwives, 60% (36/60) stated that training had changed their practice (Table 4).

**Table 4:**
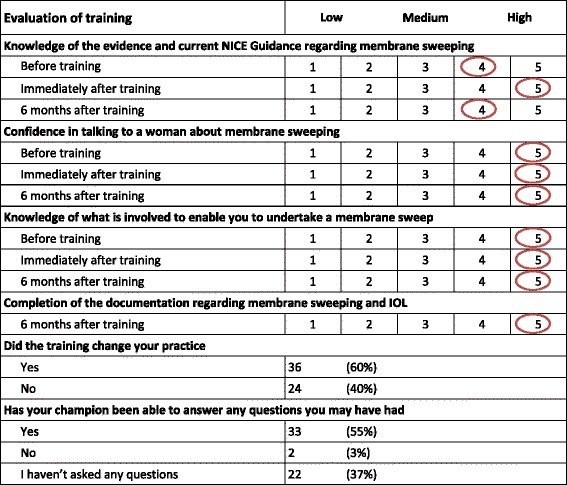
Overall evaluation of training

A sensitivity analysis investigated the impact of the additional level of clustering (teams nested within trusts) by including trust as a fixed effect but results were not sensitive to this additional clustering (results not included). The discrepancy between the ratio of the two proportions and the RR presented in the table (RR = 0.9) arises because the RR presented in the table is adjusted for time effects. In fact, it is also important to note that the ratio of the percentages swept without adjusting for time is not 0.9, but is in fact 1.05 (= intervention percentage/control percentage = 46.8/44.4). That is to say, the raw results suggest that on implementation the point estimate of the percentage of women being swept increased (from 44.4% to 46.8%), hence an increased ‘risk’ of being swept. However, in actual fact, in those clusters and time periods yet to be exposed to the intervention there was an underlying secular trend (Additional file 3: Figure S3). However, after adjusting for the underlying secular trend, we demonstrate a reversal of the treatment effect. Although of note, all these changes are small and not statistically significant. We did not examine time by treatment interactions as the study was underpowered for this comparison and this analysis had not been pre-specified.

## Discussion

The delivery of the bespoke training package to midwifery teams had little effect on the number of women being offered and accepting membrane sweeping. Had this robust evaluation not been undertaken it may well have been felt that the training had been effective and practice had changed due to the positive feedback from the midwives and evaluations such as this should be encouraged.

Studies such as these highlight the complexity of changing practice. While we did attempt to address the issues highlighted by the midwives as problematic, it is clear that this was not enough to increase the numbers of women being offered and accepting membrane sweeping. As described earlier, some of the concerns identified by the midwives were beyond the scope of the training (such as the venue) but were discussed. While we did identify a sweeping champion within each team, to provide leadership so important in change, there was no mechanism in place to enable data on sweeping to be regularly fed back to teams, which may have been helpful. While a leaflet was developed for women, there is evidence that where evidence based information is prioritised over women’s or healthcare professionals’ experiential knowledge, there is potential conflict [9], and it is plausible that not all women received the leaflet as intended.

The training session was delivered at the team meetings and in a way most suited to adult learning with discussion encouraged. The training session was delivered to the majority of team members (67%), although this did vary between the Trusts with 65% attending at BWNFT and 72% at BHH. Midwives who could not attend the team training session were trained by the champions in their team. The average size of the teams was markedly different (14 in BWNFT and seven in BHH) and it is interesting to note that the Trust with the larger teams and slightly lower attendance rates demonstrated a higher level of sweeping, although no improvement was seen overall (we did not test whether this was statistically significant). While this attempt to change practice had high-quality evidence to underpin it, reducing the rate of induction of labour would not directly affect the community midwives undertaking the sweeping. The direct effect would have been felt by the women themselves and the Labour Wards in the Trusts and it is plausible that this influenced whether the midwives did change their practice.

### Strengths and limitations

This study provides robust evidence of the effect of a bespoke training package on improving implementation of NICE Guidance to undertake membrane sweeping to reduce induction of labour and such evaluations are relatively uncommon. A recent review [10] suggests that there have been advancements in factors that influence training effectiveness and transfer of training but that robust evaluation should be encouraged and evaluation of methods of getting evidence into practice is essential to informing good quality care. One of the main obstacles to evaluating such interventions, or change, is the lack of opportunity to randomise: evidence of effectiveness is not required before implementation and so changes are often instigated before evaluation. Evaluation after instigation can be possible, either by comparison with other providers who have not instigated change, or with service provision before the change occurred, or with both before and control comparisons, but it is well-known that this forms lower-quality evidence. Stepped wedge randomised trials have been suggested as a pragmatic and appropriate option for evaluation of service delivery type interventions [11]. While this design has been recommended in service evaluations, it is important to ensure appropriate input from an experienced statistician due to the complex nature of the design and ensuing data analysis. Both BWNFT and BHH planned to sequentially roll out a training module and collaboration with the CLAHRC researchers at the University of Birmingham made this evaluation possible. Data collection was relatively complete for the trial, thus increasing reliability and validity. We did not allow for any multiplicity of outcomes in our power calculation. While the primary outcome, sweeping, has been reported in two different ways (number of sweeps and proportion of women swept), these two outcomes are very highly correlated and any multiplicity correction would be highly conservative.

We observed a significant underlying temporal trend in the proportion of women offering a membrane sweep over the duration of the study period. There are a number of possible explanations for this. Contamination between teams is a possibility, but unlikely as the teams did not mix regularly. It is more likely that the very movement that lead local Trust decision-makers to initiate this training package, also penetrated down to the teams and the midwives, akin to a rising tide [12].

One limitation of the study is that data regarding membrane sweeping were collected from the hospital notes, as described earlier, and it could be argued that this does not reflect actual practice. While it is possible, it was felt to be very unlikely that a membrane sweep would be undertaken and not recorded in the notes. The data were extracted by the same data clerk, blind to the date of training of the team and training was given by clinical midwives as to where this would be recorded.

A method for characterising and designing behaviour change interventions, which includes the ‘COM-B’ system, has been developed by Michie et al. [13], and it may be that use of such a systematic approach would have improved the number of women being offered and accepting membrane sweeping. The ‘COM-B’ system provides a framework for understanding behaviour with three essential conditions interacting to generate change, which are capability, motivation and opportunity. Capability is defined as the individual’s psychological and physical capacity to engage in the activity concerned and it includes having the necessary knowledge and skills. Motivation is defined as all those brain processes that energize and direct behaviour, not just goals and conscious decision-making. It includes habitual processes, emotional responses, as well as analytical decision-making. Opportunity is defined as all the factors that lie outside the individual that make the behaviour possible or prompt it.

Achieving and maintaining behaviour change remains challenging and Michie [14] suggests that meeting it requires a systematic method for analysing the target behaviours as a starting point for designing an intervention; selecting interventions most likely to be effective; publishing details of interventions in trial protocols to enable accurate replication and evidence synthesis and drawing on relevant theory to guide both the intervention design and evaluation. Such knowledge and skills may not be accessible to the majority of healthcare providers. Nonetheless, use of such methods should be encouraged.

## Conclusion

Novel ways of evaluating service change to improve uptake of NICE Guidance should be encouraged and use of the stepped wedge design offers a pragmatic and useful methodology in such situations, even if results showed no significant difference in this instance. In the future, use of a systematic approach to the development of behaviour change interventions should be encouraged to increase the likelihood of success and results should be fed back to Trusts to further encourage collaboration and change.

## Additional files

Additional file 1: Figure S1. Trial design. (DOCX 594 kb)

Additional file 2: Table S1. Maternal characteristics. (DOCX 16 kb)

Additional file 3: Figure S3. Demonstration of the underlying secular trend of the numbers of women having a membrane sweep over the weeks of the study. (DOCX 79 kb)

## Abbreviations

BHH: Birmingham Heartlands Hospital
BWNFT: Birmingham Women’s NHS Foundation Trust
CLAHRC BBC: Collaboration for Leadership and Applied Health Research and Care Birmingham and Black Country
ICC: Inter cluster correlation
NHS: National Health Service
NICE: National Institute for Health and Care Excellence
R&D: Research and Development
