# Supplementary figures and images for: Evaluation of a bespoke training to increase uptake by midwifery teams of NICE Guidance for membrane sweeping to reduce induction of labour: a stepped wedge cluster randomised design
Source: Trials. 2017 Jul 27;18:357. doi: 10.1186/s13063-017-2106-1 (PMC5530942; doi:10.1186/s13063-017-2106-1)

**Supplementary Figure 1 - Stepped wedge cluster randomised trial design**


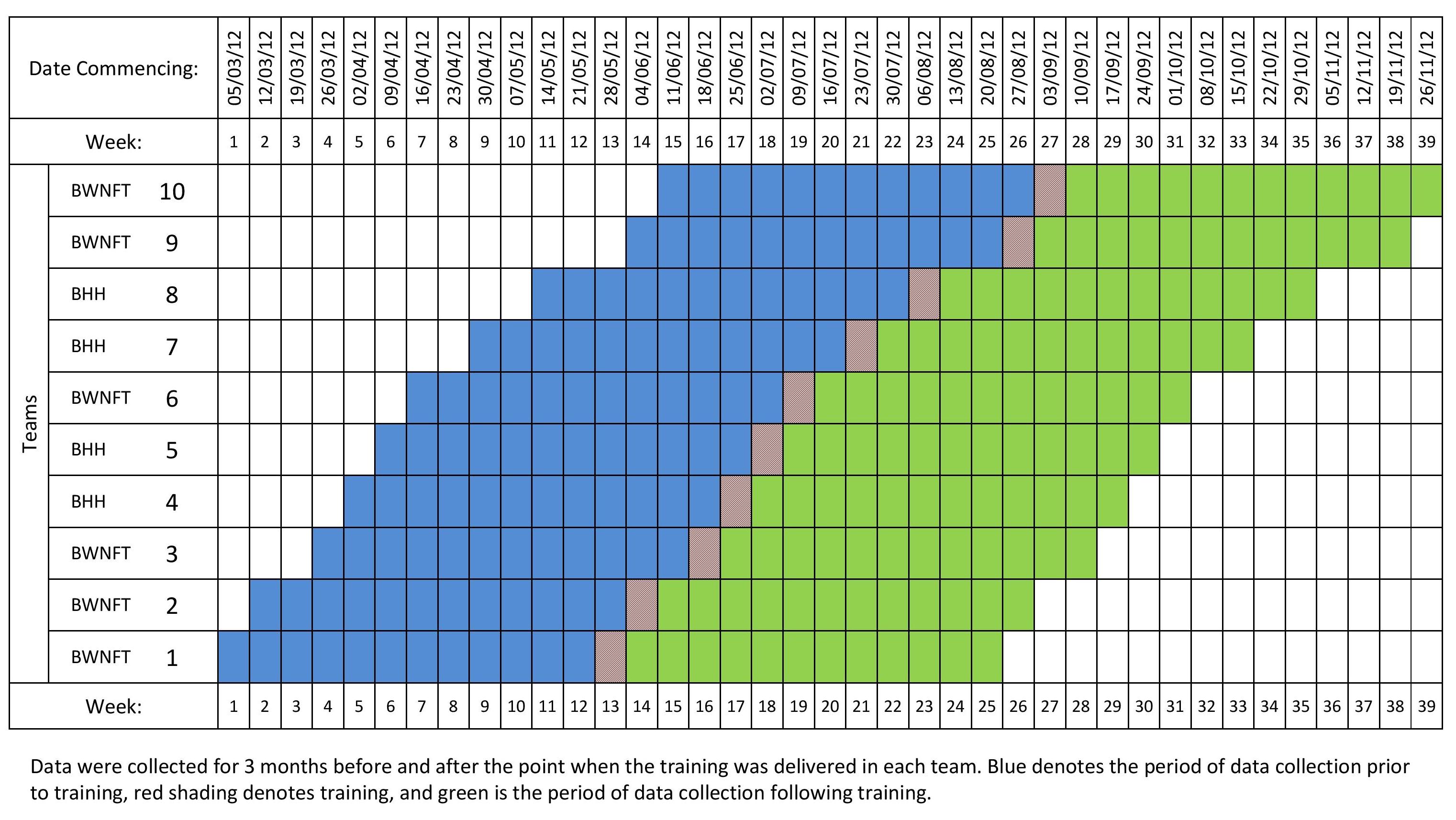

Supplement: Supplementary file 1 — Trial design. (DOCX 594 kb) [file 13063_2017_2106_MOESM1_ESM.docx]
