# Supplementary material for: Evaluation of a bespoke training to increase uptake by midwifery teams of NICE Guidance for membrane sweeping to reduce induction of labour: a stepped wedge cluster randomised design
Source: Trials. 2017 Jul 27;18:357. doi: 10.1186/s13063-017-2106-1 (PMC5530942; doi:10.1186/s13063-017-2106-1)
Supplement: Supplementary file 2 — Maternal characteristics. (DOCX 16 kb) [file 13063_2017_2106_MOESM2_ESM.docx]

**Table S1 – Comparison of the influence of maternal characteristics on sweeping before and after training**

| **Influence of** | **Before training**  **n=1417** | **After training**  **n=1356** | **Relative Risk (95% CI)** | **P-value** |
| --- | --- | --- | --- | --- |
| **Women’s age (years)** | | | | |
| <20 years | 39/77 (50.6%) | 40/81 (49.4%) | 1.13 (0.57, 2.24) | 0.74 |
| 20-35 years | 488/1124 (43.4%) | 486/1064 (45.7%) | 0.79 (0.61, 1.03) | 0.08 |
| >35 years | 102/216 (47.2%) | 108/211 (51.2%) | 1.11 (0.74, 1.67) | 0.62 |
| **Parity** | | | | |
| Nulliparous | 363/849 (42.8%) | 345/788 (43.8%) | 0.92 (0.68, 1.24) | 0.60 |
| Multiparous | 266/568 (46.8%) | 289/568 (50.9%) | 0.88 (0.64, 1.21) | 0.45 |
| **Ethnicity** | | | | |
| Africa | 27/89 (30%) | 33/81 (41%) | 1.05 (0.49, 2.28) | 0.90 |
| Asia – South | 151/442 (34%) | 126/427 (30%) | 0.76 (0.51, 1.15) | 0.19 |
| Asia – Other | 5/16 (31%) | 9/19 (47%) | 0.80 (0.18, 3.58) | 0.77 |
| Caribbean | 20/60 (33%) | 21/57 (37%) | 0.95 (0.37, 2.41) | 0.91 |
| European – Britain | 359/642 (56%) | 384/619 (62%) | 0.99 (0.79, 1.24) | 0.91 |
| European – Other | 24/65 (37%) | 24/55 (44%) | 1.06 (0.44, 2.56) | 0.90 |
| Middle East | 11/43 (26%) | 11/47 (23%) | 1.24 (0.41, 3.74) | 0.70 |
| Other | 32/60 (53%) | 23/42 (55%) | 1.11 (0.48, 2.54) | 0.81 |
| **BMI** | | | | |
| ≤18 | 25/57 (43.9%) | 24/54 (44.4%) | 0.84 (0.35, 2.00) | 0.69 |
| 19-34 | 554/1251 (44.3%) | 561/1189 (47.2%) | 0.87 (0.68, 1.11) | 0.25 |
| ≥35 | 50/108 (46.3%) | 48/111 (43.2%) | 0.99 (0.53, 1.86) | 0.98 |
| **Index of multiple deprivation from postcode** | | | | |
| Quintile 1 | 364/908 (40.1%) | 338/845 (40.0%) | 0.90 (0.67, 1.20) | 0.46 |
| Quintile 2 | 121/244 (49.6%) | 143/252 (56.7%) | 0.88 (0.60, 1.30) | 0.52 |
| Quintile 3 | 102/191 (53.4%) | 93/158 (58.9%) | 1.02 (0.67, 1.54) | 0.93 |
| Quintile 4 | 31/52 (59.6%) | 44/67 (65.7%) | 1.29 (0.62, 2.66) | 0.50 |
| Quintile 5 | 11/20 (55.0%) | 16/32 (50.0%) | 1.20 (0.34, 4.24) | 0.77 |

Analysis of outcomes excludes those with missing outcome data
