# Supplementary material for: Evaluation of a bespoke training to increase uptake by midwifery teams of NICE Guidance for membrane sweeping to reduce induction of labour: a stepped wedge cluster randomised design
Source: Trials. 2017 Jul 27;18:357. doi: 10.1186/s13063-017-2106-1 (PMC5530942; doi:10.1186/s13063-017-2106-1)

**Figure S3 Demonstration of the underlying secular trend of the numbers of women having a membrane sweep over the weeks of the study**


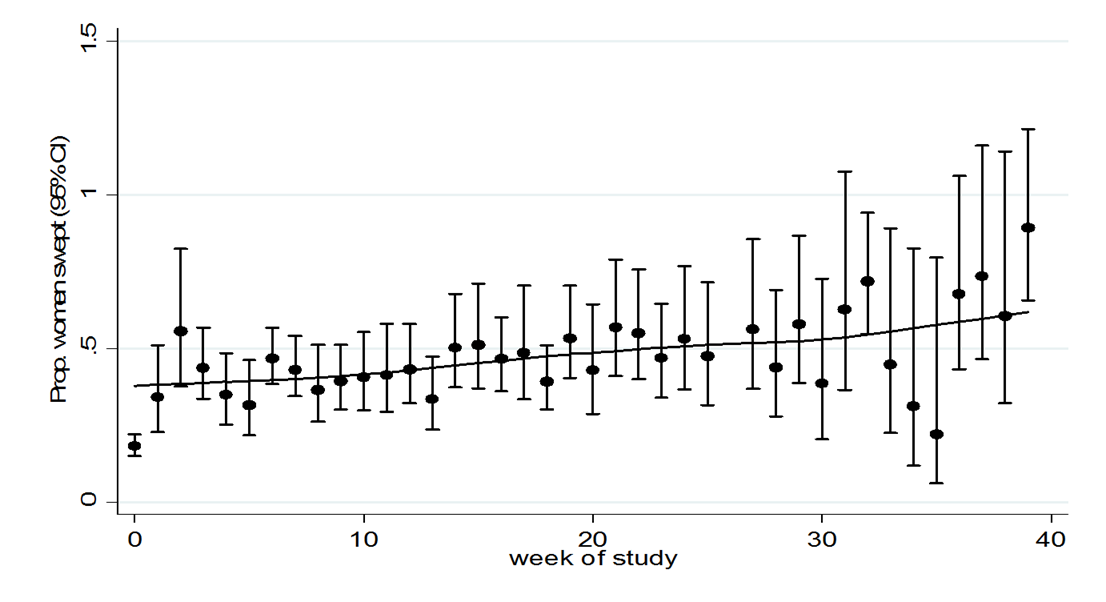

Supplement: Supplementary file 3 — Demonstration of the underlying secular trend of the numbers of women having a membrane sweep over the weeks of the study. (DOCX 79 kb) [file 13063_2017_2106_MOESM3_ESM.docx]
